# Symptom-Only Localization of Brainstem Ischemia Using Large Language Models Versus Neurologists in Diffusion-Weighted Imaging–Positive Cases: Retrospective Single-Center Study

**DOI:** 10.2196/87163

**Published:** 2026-07-08

**Authors:** Nedim Beste, Thomas Dratsch, Jonathan Kottlors, Pia Floßdorf, Agni-Maria Konitsioti, Lukas J Volz, Uta Hanning, Daniel Pinto dos Santos, Lukas Goertz, David Zopfs, Christoph Kabbasch, Marc Schlamann, Kai Roman Laukamp, Michael Schönfeld

**Affiliations:** 1 University Hospital Cologne, Kerpener Str 62, Cologne, 50937, Germany, 49 2214780; 2 University Medical Centre Mannheim, Mannheim, Germany; 3 Johannes Gutenberg University Mainz, Mainz, Germany

**Keywords:** brainstem ischemia, GPT-4, diagnostic accuracy, language models, stroke localization, artificial intelligence, AI

## Abstract

**Background:**

Symptom-based localization of brainstem ischemia is challenging because of the anatomical complexity of the brainstem and the nonspecific overlap of clinical syndromes. Whether large language models (LLMs) can meaningfully assist in this task remains uncertain.

**Objective:**

This study aimed to compare the performance of several OpenAI LLMs and neurologists in localizing diffusion-weighted imaging (DWI)–confirmed brainstem ischemic lesions based on symptom descriptions alone.

**Methods:**

In this retrospective single-center study, 109 patients with DWI-confirmed acute brainstem ischemia were included. Three neurologists and 6 LLMs (GPT-5, GPT-4, GPT-4.1, GPT-4o, o3, and o3-pro) predicted lesion localization using a combined anatomical-lateral end point (left or right midbrain, pons, and medulla) based on symptom descriptions alone. Overall and regional accuracy, the Cohen κ, 6-class confusion matrices, and point-biserial correlations between symptom count and correct prediction were assessed. Because all raters evaluated the same cases, paired McNemar tests with Benjamini-Hochberg correction were used for pairwise performance comparisons.

**Results:**

GPT-4 and GPT-4o achieved the highest overall accuracy (61/109, 56%; 95% CI 46.1%‐65.5%). Agreement with the DWI reference standard remained limited across all raters, with the Cohen κ reaching a maximum of 0.291 for GPT-4o. Confusion matrices showed that higher performance was driven mainly by pontine cases, whereas misclassification remained frequent in mesencephalic and medullary lesions. Regional analyses outside the pons were imprecise because mesencephalic and medullary subgroups each contained only 16 cases. A higher number of documented symptoms was associated with correct prediction for GPT-4, GPT-5, GPT-o3, and 1 neurologist.

**Conclusions:**

Although some LLMs showed higher relative accuracy than the participating neurologists, absolute performance remained limited and clinically insufficient. These findings are best interpreted as an exploratory benchmark under constrained conditions: absolute performance remained modest, agreement beyond chance was limited, and performance outside pontine lesions was inconclusive.

## Introduction

### Challenges in Clinical Localization of Brainstem Ischemia

The accurate detection of ischemic strokes in the brainstem presents a significant challenge in neurology, given the complexity of this region’s anatomy and the variability of clinical symptoms. Even experienced neurologists may struggle, as similar syndromes can result from lesions in multiple closely adjacent structures. As a result, accurate localization often remains elusive without advanced imaging modalities [1].

### Advanced Imaging Techniques and the Limits of Symptom-Based Diagnosis

Advanced imaging techniques, especially diffusion-weighted imaging (DWI), have improved the identification of ischemic lesions in the brainstem, offering more sensitive diagnostic approaches with superior sensitivity compared to traditional T2-weighted magnetic resonance imaging. Studies indicate that combining axial and coronal DWI sequences further improves the detectability of small lesions, significantly enhancing diagnostic confidence and guiding clinical management [2,3]. However, the accuracy of clinical localization without imaging remains limited.

### Large Language Models as Emerging Supporting Tools for Clinical Reasoning

Large language models (LLMs), particularly the GPT series by OpenAI, have demonstrated utility in medical diagnostics by identifying patterns within clinical narratives, supporting diagnostic reasoning, and generating differential diagnoses by leveraging vast text-based training data [4,5]. While their overall performance is strongest when structured and multimodal data are available, they can outperform or complement expert clinicians in specific constrained tasks, though real-world accuracy for complex cases remains a topic of ongoing research [6,7]. However, their effectiveness in localizing acute brainstem ischemia based solely on clinical symptoms has not been clearly established [8]. Specifically, evidence for LLM application to stroke-specific neurolocalization tasks remains sparse, particularly when predictions rely on symptom descriptions alone [9].

### Objectives of the Study

The objective of this study was to compare the diagnostic accuracy of several OpenAI LLMs (GPT-5, GPT-4, GPT-4.1, GPT-4o, GPT-o3, and GPT-o3 pro) with that of neurologists in localizing brainstem ischemic lesions based on clinical symptoms alone. Additionally, we explored whether enhanced “reasoning” prompts could improve diagnostic accuracy.

## Methods

### Ethical Considerations

This retrospective single-center study was approved by the local ethics committee (Ethics Committee of the Physician Board Hamburg; WF 14/17). The requirement for informed consent was waived because of the retrospective design and use of previously collected clinical data. All data used for analysis were deidentified prior to evaluation. No participants received compensation. No identifiable individual participant information or images are included in the manuscript or supplementary material.

### Study Design and Participants

This study was designed to align with the Standards for Reporting Diagnostic Accuracy Studies (STARD) checklist [10]. We retrospectively analyzed the data of 152 patients hospitalized between January 2013 and September 2020 diagnosed with brainstem ischemia that was visible on DWI. After exclusion of patients exhibiting DWI-positive ischemia in more than one brainstem region or in other regions of the brain than the brainstem, 109 patients (77/109, 70.6% male and 32/109, 29.4% female; mean age 66.6, SD 11.3 years; range 45.0‐90.0 years) were included in the final analysis. Written records of the neurological examination upon admission were available for all patients. For each patient, a median of 6.0 (IQR 4.0-7.0) symptoms was available (range 3.0‐13.0). Localization of ischemic lesions on DWI was validated by a neuroradiologist (MS). DWI-lesions consistent with brainstem ischemia were prevalent in the left mesencephalon in 4.6% (5/109), in the right mesencephalon in 10.1% (11/109), in the left pons in 33% (36/109), in the right pons in 37.6% (41/109), in the left medulla in 7.3% (8/109), and in the right medulla in 7.3% (8/109). There was no missing data in the dataset.

### LLM Query Settings

Model access method was via OpenAI application programming interface. The exact prompting setup can be found in Multimedia Appendix 1. All queries were conducted in single run format. Temperature (ie, grade of deterministic responses) was set to minimum (0).

### Performance Comparison

GPT-5, GPT-4, GPT-4.1, GPT-4o, GPT-o3, GPT-o3 pro, and 3 neurologists (with 2, 11, and 14 years of experience) were tasked with predicting the most likely location of the brainstem ischemia based on each patient’s symptoms at the time of the magnetic resonance imaging. GPT-4, GPT-4.1, GPT-4o, GPT-5, and GPT-o3 are transformer-based LLMs developed by OpenAI. While GPT-4 is known to contain approximately 175 billion parameters, OpenAI has not disclosed the architecture or size of the subsequent models. GPT-4.1 and GPT-4o represent optimized versions with improved stability and multiturn reasoning, while GPT-5 is designed to offer more advanced contextual understanding. GPT-o3 and GPT-o3 pro are reasoning-augmented variants aiming to enhance step-by-step clinical inference [11]. For this study, all models were prompted using chain-of-thought prompting, where a model is tasked with elaborating its reasoning process, as this process has been shown to result in more accurate output of LLMs; importantly, the reasoning models GPT-o3 and GPT-o3 pro were provided a tailored prompt (exact prompts provided in Multimedia Appendix 1) [12]. To address the unexpectedly low performance of GPT-o3 pro, raw model outputs were reviewed for potential parsing or extraction errors. No evidence was found that valid responses had been systematically missed by the evaluation pipeline. Clinical symptom lists were extracted from the admission records by members of the study team and standardized into a uniform symptom-only format before evaluation. All raters evaluated the same curated symptom information extracted from the clinical records; no additional imaging information beyond the final DWI-based reference standard was provided for prediction.

### Statistical Analysis

Overall diagnostic accuracy and regional accuracy by brainstem compartment were calculated for all LLMs and neurologists. Because all raters evaluated the same 109 cases, paired comparisons of diagnostic accuracy were performed using McNemar tests, and *P* values were adjusted for multiple testing using the Benjamini-Hochberg procedure. Agreement between predicted and reference lesion location was assessed using the Cohen κ. The association between the number of documented symptoms and the correctness of prediction was assessed using point-biserial correlation, given the binary outcome (correct or incorrect). Confusion matrices were generated for each rater using the combined anatomical-lateral end point (left or right mesencephalon, pons, and medulla oblongata). To ensure consistency with the overall accuracy values reported, all 109 cases were retained; predictions that could not be mapped to one of the 6 predefined classes were assigned to an additional category, “invalid or missing prediction.” All analyses were performed in R (version 4.2.1; R Foundation for Statistical Computing) using the packages readxl, dplyr, tidyr, ggplot2, gt, yardstick, broom, tibble, and scales. Complete paired McNemar comparisons of overall and regional accuracy are provided in Multimedia Appendix 2 and Multimedia Appendix 3, respectively. Class-wise prediction distributions are provided in Multimedia Appendix 4.

## Results

### Overall Accuracy

**Figure 1.**
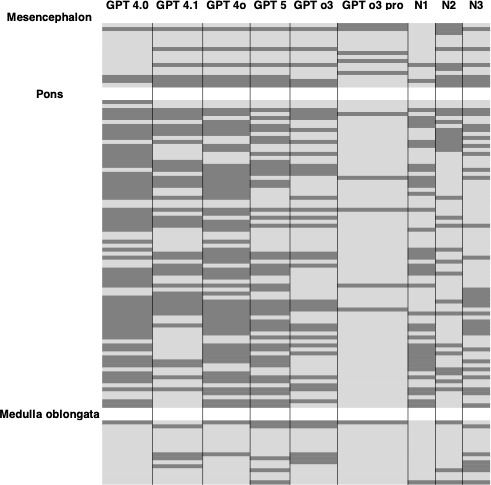
Prediction results by anatomical region and large language models (LLMs) and neurologists. Each row represents an individual patient with diffusion-weighted imaging–confirmed brainstem ischemia; columns show predictions from LLMs and neurologists. Dark gray indicates correct and light gray indicates incorrect predictions. GPT-4 and GPT-4o achieved the highest accuracy in pontine infarcts, whereas performance was lower in midbrain and medullary lesions. N1: neurologist 1; N2: neurologist 2; N3: neurologist 3.

GPT-4 and GPT-4o achieved the highest overall accuracy, each reaching 56% (61/109; 95% CI 46.1%‐65.5%). GPT-5 reached 48.6% (53/109; 95% CI 38.9%‐58.4%) and GPT-4.1 reached 41.3% (45/109; 95% CI 31.9%‐51.1%). In paired McNemar comparisons, GPT-4 and GPT-4o significantly outperformed GPT-o3 pro and several neurologists after Benjamini-Hochberg correction. GPT-o3 pro showed the lowest overall performance (11/109, 10.1%; 95% CI 5.2%‐17.3%; Table 1 and Figure 1). Full paired McNemar comparisons, including discordant counts, favored-rater direction, McNemar chi-square statistics, and Benjamini-Hochberg–adjusted *P* values, are shown in Multimedia Appendix 2.

**Table 1.** Performance for the prediction of diffusion-weighted imaging lesions in total and split by brain stem regions (N=109). Full pairwise comparisons are presented in MultimediaAppendices 2  3.

	Brainstem location			
	Mesencephalon, n/N (%; 95% CI)	Pons, n/N (%; 95% CI)	Medulla, n/N (%; 95% CI)	Total, n/N (%; 95% CI)
GPT-4	3/16 (18.8; 4.0‐45.6)	57/77 (74.0; 62.8‐83.4)	1/16 (6.3; 0.2‐30.2)	61/109 (56.0; 46.1‐65.5)
GPT-4.1	6/16 (37.5; 15.2‐64.6)	35/77 (45.5; 34.1‐57.2)	4/16 (25.0; 7.3‐52.4)	45/109 (41.3; 31.9‐51.1)
GPT-4o	6/16 (37.5; 15.2‐64.6)	53/77 (68.8; 57.3‐78.9)	2/16 (12.5; 1.6‐38.3)	61/109 (56.0; 46.1‐65.5)
GPT-5	6/16 (37.5; 15.2‐64.6)	42/77 (54.5; 42.8‐65.9)	5/16 (31.2; 11.0‐58.7)	53/109 (48.6; 38.9‐58.4)
GPT-o3	5/16 (31.2; 11.0‐58.7)	28/77 (36.4; 25.7‐48.1)	5/16 (31.2; 11.0‐58.7)	38/109 (34.9; 26.0‐44.6)
GPT-o3 pro	5/16 (31.2; 11.0‐58.7)	5/77 (6.5; 2.1‐14.5)	1/16 (6.3; 0.2‐30.2)	11/109 (10.1; 5.1‐17.3)
Neurologist 1	2/16 (12.5; 1.6‐38.3)	37/77 (48.1; 36.5‐59.7)	1/16 (6.3; 0.2‐30.2)	40/109 (36.7; 27.7‐46.5)
Neurologist 2	9/16 (56.2; 29.9‐80.2)	23/77 (29.9; 20.0‐41.4)	3/16 (18.8; 4.0‐45.6)	35/109 (32.1; 23.5‐41.7)
Neurologist 3	6/16 (37.5; 15.2‐64.6)	28/77 (36.4; 25.7‐48.1)	6/16 (37.5; 15.2‐64.6)	40/109 (36.7; 27.7‐46.5)

### Regional Accuracy

Significant performance differences were concentrated in pontine lesions, which represented the majority class in the cohort (77/109, 70.6%). GPT-4 achieved 74% (57/77; 95% CI 62.8%‐83.4%) and GPT-4o 68.8% (53/77; 95% CI 57.3%‐78.9%) in pontine cases. In contrast, mesencephalic and medullary subgroup analyses remained imprecise because each subgroup included only 16 cases, resulting in wide CIs. Accordingly, comparisons outside the pons should be interpreted as inconclusive rather than as evidence of equivalent performance (Table 1 and Figure 1). Furthermore, 6-class confusion matrices confirmed that the observed performance differences were driven mainly by pontine cases and made class-specific misclassification patterns transparent. Region-specific paired McNemar comparisons are shown in Multimedia Appendix 3.

### Cohen κ and Confusion Matrices

Agreement with the DWI reference standard remained limited across all raters. The Cohen κ was highest for GPT-4o (κ=0.291), followed by GPT-4.1 (κ=0.246), GPT-5 (κ=0.220), and GPT-4 (κ=0.212), whereas neurologists showed only slight agreement (κ=0.108‐0.151). Six-class confusion matrices and prediction distributions showed that the observed performance differences were driven mainly by the pontine categories, while misclassification remained frequent in mesencephalic and medullary lesions (Table 2). Class-wise prediction distributions are shown in Multimedia Appendix 4 to illustrate potential preference for the dominant pontine classes and the frequency of invalid or unmappable outputs.

**Table 2.** Agreement with actual diffusion-weighted imaging lesion locations.

Rater	Cohen κ
GPT-4	0.212
GPT-4.1	0.246
GPT-4o	0.291
GPT-5	0.220
GPT-o3	0.123
GPT-o3 pro	0.07
Neurologist 1	0.108
Neurologist 2	0.151
Neurologist 3	0.123

### Correlation With Number of Reported Symptoms

A higher number of documented symptoms was associated with correct prediction for GPT-4 (point-biserial *r*=0.281; *P*=.003), GPT-5 (point-biserial *r*=0.255; *P*=.007), GPT-o3 (point-biserial *r*=0.226; *P*=.02), and neurologist 1 (point-biserial *r*=0.290; *P*=.002). No statistically significant associations were observed for GPT-4.1, GPT-4o, GPT-o3 pro, neurologist 2, or neurologist 3 (Table 3).

**Table 3.** Correlation of models with number of reported symptoms.

Rater	Point-biserial correlation coefficient *r*	*P* value
GPT-4	0.281	.003
GPT-4.1	0.135	.16
GPT-4o	0.153	.11
GPT-5	0.255	.007
GPT-o3	0.226	.02
GPT-o3 pro	0.093	.34
Neurologist 1	0.292	.002
Neurologist 2	0.069	.48
Neurologist 3	0.095	.33

## Discussion

### Principal Results

We found that GPT-4 and GPT-4o achieved the highest overall accuracy, exceeding the neurologists’ performance. However, despite superior relative performance, GPT-4 and GPT-4o still misclassified approximately 44% of cases. Performance was especially poor in the midbrain and medulla categories. This indicates that the overall clinical utility of these models remains limited in their current form. As such, they cannot be recommended as diagnostic tools for this specific task in isolation.

### Interpretation of Model-Specific Differences

The combined interpretation of raw accuracy, the Cohen κ and 6-class confusion matrices suggests that the apparent advantage of GPT-4 and GPT-4o should be interpreted cautiously. While these models achieved the highest overall accuracy, agreement with the DWI reference standard remained only fair at best, and the confusion matrices indicate that performance was driven largely by the dominant pontine classes rather than by uniformly reliable localization across all anatomical-lateral categories. This limits the clinical significance of the observed superiority and argues against overinterpreting these findings as evidence of current diagnostic utility.

Interestingly, the more recent GPT-5 and GPT-4.1 models underperformed compared with GPT-4 and GPT-4o, and the reasoning-enhanced models (o3 and o3-pro) performed substantially worse, with o3-pro reaching only 10% accuracy. This counterintuitive result suggests that increasing reasoning depth via prompting does not improve performance when the available information (ie, symptoms alone) is not sufficiently specific to support a valid inference. Overinterpretation or spurious associations may have led to incorrect predictions. This observation aligns with prior reports on prompt engineering pitfalls in clinical LLM applications [9,13,14].

However, GPT-4.1 demonstrated a relatively higher agreement with true lesion locations despite its lower overall accuracy compared with GPT-4 and GPT-4o. This indicates that, while GPT-4.1 made more incorrect predictions overall, its correct answers were better distributed across all anatomical classes, leading to a higher-than-expected agreement beyond chance. In contrast, GPT-4 and GPT-4o tended to favor more frequent lesion locations (eg, pons), which may have inflated raw accuracy at the expense of true localization diversity. This highlights the importance of using multiple evaluation metrics when assessing clinical prediction models.

### Role of Clinical Input and Predictive Limits

There was a modest but statistically significant correlation between the number of documented symptoms and prediction accuracy for GPT-4, GPT-o3, and one neurologist. This suggests that prediction reliability may improve with richer clinical input.

Our findings also support the assumption that localizing brainstem ischemia based solely on clinical symptoms is inherently difficult. The brainstem contains numerous compact and overlapping functional pathways. Lesions in different locations can produce similar symptoms, and conversely, small lesions can cause complex syndromes depending on the affected tracts. This complexity likely limits the ceiling of achievable accuracy for both human and machine prediction based solely on symptoms [15-17].

### Limitations

This study has several limitations. The retrospective design introduces potential biases related to documentation and selection. All models were evaluated using written clinical information; it is possible that neurologists would perform better in real clinical interactions, where subtle signs and context might be integrated into their reasoning. Although all raters received the same curated symptom information, conversion of clinical records into standardized symptom lists may itself have reduced ecological validity compared with real-world bedside assessment. In addition, we only tested 3 neurologists; broader expert benchmarking could yield different comparative results. Also, the brainstem ischemia localization was confirmed by one neuroradiologist without interrater assessment.

Furthermore, the study evaluated LLMs without fine-tuning or integration of multimodal input. LLMs were used in a zero-shot setting with standardized prompts. In future work, it may be possible to improve diagnostic performance by combining LLMs with imaging data or structured clinical scores, which has shown benefit in other medical domains [18-20]. Similarly, reinforcement learning or few-shot adaptation using expert-labeled cases as well as using the median of repeated runs might improve model robustness and accuracy. An additional limitation is that the prompting strategy differed between the standard and reasoning-oriented models. Accordingly, the lower performance of the reasoning-oriented models should not be interpreted as evidence that reasoning augmentation is inherently inferior, but rather as the combined effect of model behavior and prompt design. Because GPT-o3 pro showed unexpectedly low performance, raw outputs were spot-checked for potential parsing errors. This review did not suggest a systematic extraction failure, although a subtle technical artifact cannot be fully excluded.

### Conclusions

In conclusion, some GPT-based models achieved higher relative accuracy than the participating neurologists in this constrained benchmark. However, absolute accuracy remained modest, and reasoning-augmented prompting did not improve results. These findings highlight current limitations rather than readiness for clinical implementation.

## Supplementary material

10.2196/87163 Multimedia Appendix 1 Prompt templates used for standard and reasoning-oriented models.

10.2196/87163 Multimedia Appendix 2 Pairwise McNemar comparisons of overall diagnostic accuracy.

10.2196/87163 Multimedia Appendix 3 Pairwise McNemar comparisons of regional diagnostic accuracy.

10.2196/87163 Multimedia Appendix 4 Distribution of predicted anatomical-lateral classes across raters.

## Abbreviations

API: application programming interface
DWI: diffusion-weighted imaging
LLM: large language model
STARD: Standards for Reporting Diagnostic Accuracy Studies
