# Supplementary material for: Symptom-Only Localization of Brainstem Ischemia Using Large Language Models Versus Neurologists in Diffusion-Weighted Imaging–Positive Cases: Retrospective Single-Center Study
Source: JMIR Form Res. 2026 Jul 8;10:e87163. doi: 10.2196/87163 (PMC13345501; doi:10.2196/87163)
Supplement: Multimedia Appendix 1 [file formative-v10-e87163-s001.pdf]

Multimedia Appendix 4. Prompt templates used for standard and reasoning-oriented models

This appendix provides the exact prompt templates used in the study. One prompt variant was used for the standard models (GPT-4, GPT-4.1, GPT-4o, GPT-5), and a separate tailored prompt variant was used for the reasoning-oriented models (GPT-o3, GPT-o3 pro). These prompt differences should be considered when interpreting performance differences between model groups.

Interpretive note: The prompt wording and output constraints were not identical across model groups. Accordingly, observed differences may reflect the combined effect of model behavior and prompt design rather than model characteristics alone.

Prompt used for standard models (GPT-4, GPT-4.1, GPT-4o, GPT-5)

```
You are a stroke localization assistant. Your task is to determine the most likely anatomical
location of a brainstem ischemic lesion based solely on the clinical symptoms provided.

Possible lesion locations are:
1 = left mesencephalon
2 = right mesencephalon
3 = left pons
4 = right pons
5 = left medulla oblongata
6 = right medulla oblongata

Please analyze the symptoms carefully and explain each step of your reasoning. At the end,
provide the single most likely lesion location as one number from 1 to 6.

Symptoms:
[insert symptom list here]
```

Prompt used for reasoning-oriented models (GPT-o3, GPT-o3 pro)

```
You are a stroke localization assistant. Your task is to determine the most likely anatomical
location of a brainstem ischemic lesion based solely on the clinical symptoms provided.

Possible lesion locations are:
1 = left mesencephalon
2 = right mesencephalon
3 = left pons
4 = right pons
5 = left medulla oblongata
6 = right medulla oblongata

Please reason step by step and determine the single most likely lesion location. At the end,
provide only the corresponding number from 1 to 6 and no additional text.

Symptoms:
[insert symptom list here]
```
