# Supplementary material for: Symptom-Only Localization of Brainstem Ischemia Using Large Language Models Versus Neurologists in Diffusion-Weighted Imaging–Positive Cases: Retrospective Single-Center Study
Source: JMIR Form Res. 2026 Jul 8;10:e87163. doi: 10.2196/87163 (PMC13345501; doi:10.2196/87163)
Supplement: Multimedia Appendix 2 [file formative-v10-e87163-s002.pdf]

Supplementary Table 1. Pairwise McNemar Comparisons of Overall Diagnostic Accuracy

Pairwise comparisons were performed using McNemar tests because all raters evaluated the same cases. Values were reformatted for readability; model names use hyphenated notation.

Rater 1	Rater 2	N	R1 wrong / R2 correct, n	R1 correct / R2 wrong, n	McNemar χ^2	P	BH-adjusted P
GPT-4.0	GPT-o3 pro	109	4	54	41.40	<0.001	<0.001
GPT-4o	GPT-o3 pro	109	4	54	41.40	<0.001	<0.001
GPT-5	GPT-o3 pro	109	6	48	31.13	<0.001	<0.001
GPT-4.1	GPT-o3 pro	109	5	39	24.75	<0.001	<0.001
GPT-o3	GPT-o3 pro	109	6	33	17.33	<0.001	<0.001
GPT-o3 pro	N1	109	37	8	17.42	<0.001	<0.001
GPT-o3 pro	N3	109	37	8	17.42	<0.001	<0.001
GPT-4o	N2	109	9	35	14.21	<0.001	<0.001
GPT-o3 pro	N2	109	31	7	13.92	<0.001	<0.001
GPT-4.0	N2	109	13	39	12.02	<0.001	.002
GPT-4o	N1	109	7	28	11.43	<0.001	.002
GPT-4o	GPT-o3	109	10	33	11.26	<0.001	.002
GPT-4.0	GPT-o3	109	13	36	9.88	.002	.004
GPT-4.0	N1	109	10	31	9.76	.002	.004
GPT-4o	N3	109	10	31	9.76	.002	.004
GPT-4.0	N3	109	14	35	8.16	.004	.010
GPT-5	GPT-o3	109	6	21	7.26	.007	.015
GPT-4.1	GPT-4o	109	26	10	6.25	.012	.024
GPT-5	N2	109	14	32	6.28	.012	.024
GPT-4.0	GPT-4.1	109	12	28	5.62	.018	.032
GPT-5	N1	109	8	21	4.97	.026	.044
GPT-5	N3	109	10	23	4.36	.037	.060

Rater 1	Rater 2	N	R1 wrong / R2 correct, n	R1 correct / R2 wrong, n	McNemar χ^2	P	BH-adjusted P
GPT-4.1	GPT-5	109	17	9	1.89	.170	.262
GPT-4.1	N2	109	17	27	1.84	.175	.262
GPT-4.0	GPT-5	109	13	21	1.44	.230	.331
GPT-4.1	GPT-o3	109	10	17	1.33	.248	.331
GPT-4o	GPT-5	109	14	22	1.36	.243	.331
GPT-4.1	N3	109	14	19	0.48	.486	.625
GPT-4.1	N1	109	16	21	0.43	.511	.626
N2	N3	109	22	17	0.41	.522	.626
N1	N2	109	21	26	0.34	.560	.650
GPT-o3	N2	109	18	21	0.10	.749	.842
GPT-o3	N1	109	21	19	0.03	.874	.926
GPT-o3	N3	109	19	17	0.03	.868	.926
GPT-4.0	GPT-4o	109	11	11	0.00	1.000	1.000
N1	N3	109	21	21	0.00	1.000	1.000
