# Supplementary material for: Symptom-Only Localization of Brainstem Ischemia Using Large Language Models Versus Neurologists in Diffusion-Weighted Imaging–Positive Cases: Retrospective Single-Center Study
Source: JMIR Form Res. 2026 Jul 8;10:e87163. doi: 10.2196/87163 (PMC13345501; doi:10.2196/87163)
Supplement: Multimedia Appendix 3 [file formative-v10-e87163-s003.pdf]

Supplementary Table 2. Pairwise McNemar Comparisons of Regional Diagnostic Accuracy

Regional pairwise comparisons were performed within each brainstem compartment. Mesencephalic and medullary subgroup analyses should be interpreted cautiously because of limited subgroup size.

Region	Rater 1	Rater 2	N	R1 wrong / R2 correct, n	R1 correct / R2 wrong, n	McNemar χ^2	P	BH-adjusted P
Medulla oblongata	GPT-4.0	GPT-5	16	4	0	4.00	.045	.257
Medulla oblongata	GPT-4.0	GPT-o3	16	4	0	4.00	.045	.257
Medulla oblongata	GPT-4.0	N3	16	6	1	3.57	.059	.257
Medulla oblongata	GPT-5	GPT-o3 pro	16	0	4	4.00	.045	.257
Medulla oblongata	GPT-5	N1	16	0	4	4.00	.045	.257
Medulla oblongata	GPT-o3	GPT-o3 pro	16	0	4	4.00	.045	.257
Medulla oblongata	GPT-o3 pro	N3	16	6	1	3.57	.059	.257
Medulla oblongata	N1	N3	16	5	0	5.00	.025	.257
Medulla oblongata	GPT-4.0	GPT-4.1	16	4	1	1.80	.180	.315
Medulla oblongata	GPT-4.0	N2	16	2	0	2.00	.157	.315
Medulla oblongata	GPT-4.1	GPT-o3 pro	16	1	4	1.80	.180	.315
Medulla oblongata	GPT-4.1	N1	16	1	4	1.80	.180	.315
Medulla oblongata	GPT-4o	GPT-5	16	4	1	1.80	.180	.315
Medulla oblongata	GPT-4o	GPT-o3	16	3	0	3.00	.083	.315
Medulla oblongata	GPT-4o	N3	16	5	1	2.67	.102	.315
Medulla oblongata	GPT-5	N2	16	0	2	2.00	.157	.315
Medulla oblongata	GPT-o3	N1	16	1	5	2.67	.102	.315
Medulla oblongata	GPT-o3 pro	N2	16	2	0	2.00	.157	.315
Medulla oblongata	N1	N2	16	2	0	2.00	.157	.315
Medulla oblongata	N2	N3	16	4	1	1.80	.180	.315
Medulla oblongata	GPT-4.0	GPT-4o	16	1	0	1.00	.317	.463
Medulla oblongata	GPT-4.1	GPT-4o	16	1	3	1.00	.317	.463

Region	Rater 1	Rater 2	N	R1 wrong / R2 correct, n	R1 correct / R2 wrong, n	McNemar χ^2	P	BH-adjusted P
Medulla oblongata	GPT-4.1	N3	16	3	1	1.00	.317	.463
Medulla oblongata	GPT-4o	GPT-o3 pro	16	0	1	1.00	.317	.463
Medulla oblongata	GPT-o3	N2	16	2	4	0.67	.414	.580
Medulla oblongata	GPT-4.1	GPT-o3	16	2	1	0.33	.564	.705
Medulla oblongata	GPT-4o	N1	16	1	2	0.33	.564	.705
Medulla oblongata	GPT-4o	N2	16	2	1	0.33	.564	.705
Medulla oblongata	GPT-4.1	GPT-5	16	3	2	0.20	.655	.739
Medulla oblongata	GPT-5	N3	16	3	2	0.20	.655	.739
Medulla oblongata	GPT-o3	N3	16	3	2	0.20	.655	.739
Medulla oblongata	GPT-4.1	N2	16	3	4	0.14	.705	.772
Medulla oblongata	GPT-4.0	N1	16	1	1	0.00	1.000	1.000
Medulla oblongata	GPT-5	GPT-o3	16	2	2	0.00	1.000	1.000
Medulla oblongata	GPT-o3 pro	N1	16	1	1	0.00	1.000	1.000
Medulla oblongata	GPT-4.0	GPT-o3 pro	16	0	0		nan	nan
Mesencephalon	GPT-4.0	GPT-4.1	16	3	0	3.00	.083	.156
Mesencephalon	GPT-4.0	GPT-4o	16	3	0	3.00	.083	.156
Mesencephalon	GPT-4.0	GPT-5	16	3	0	3.00	.083	.156
Mesencephalon	GPT-4.0	N2	16	6	0	6.00	.014	.156
Mesencephalon	GPT-4.0	N3	16	3	0	3.00	.083	.156
Mesencephalon	GPT-4.1	N1	16	0	4	4.00	.045	.156
Mesencephalon	GPT-4.1	N2	16	3	0	3.00	.083	.156
Mesencephalon	GPT-4o	N1	16	0	4	4.00	.045	.156
Mesencephalon	GPT-4o	N2	16	3	0	3.00	.083	.156
Mesencephalon	GPT-5	N1	16	0	4	4.00	.045	.156

Region	Rater 1	Rater 2	N	R1 wrong / R2 correct, n	R1 correct / R2 wrong, n	McNemar χ^2	P	BH-adjusted P
Mesencephalon	GPT-5	N2	16	3	0	3.00	.083	.156
Mesencephalon	GPT-o3	N1	16	0	3	3.00	.083	.156
Mesencephalon	GPT-o3	N2	16	4	0	4.00	.045	.156
Mesencephalon	N1	N2	16	7	0	7.00	.008	.156
Mesencephalon	N1	N3	16	4	0	4.00	.045	.156
Mesencephalon	N2	N3	16	0	3	3.00	.083	.156
Mesencephalon	GPT-o3 pro	N2	16	7	3	1.60	.206	.363
Mesencephalon	GPT-4.0	GPT-o3	16	3	1	1.00	.317	.414
Mesencephalon	GPT-4.1	GPT-o3	16	0	1	1.00	.317	.414
Mesencephalon	GPT-4o	GPT-o3	16	0	1	1.00	.317	.414
Mesencephalon	GPT-5	GPT-o3	16	0	1	1.00	.317	.414
Mesencephalon	GPT-o3	N3	16	1	0	1.00	.317	.414
Mesencephalon	GPT-o3 pro	N1	16	2	5	1.29	.257	.414
Mesencephalon	GPT-4.0	GPT-o3 pro	16	4	2	0.67	.414	.518
Mesencephalon	GPT-4.0	N1	16	1	2	0.33	.564	.676
Mesencephalon	GPT-4.1	GPT-o3 pro	16	4	5	0.11	.739	.764
Mesencephalon	GPT-4o	GPT-o3 pro	16	4	5	0.11	.739	.764
Mesencephalon	GPT-5	GPT-o3 pro	16	4	5	0.11	.739	.764
Mesencephalon	GPT-o3 pro	N3	16	5	4	0.11	.739	.764
Mesencephalon	GPT-o3	GPT-o3 pro	16	4	4	0.00	1.000	1.000
Mesencephalon	GPT-4.1	GPT-4o	16	0	0		nan	nan
Mesencephalon	GPT-4.1	GPT-5	16	0	0		nan	nan
Mesencephalon	GPT-4.1	N3	16	0	0		nan	nan
Mesencephalon	GPT-4o	GPT-5	16	0	0		nan	nan

Region	Rater 1	Rater 2	N	R1 wrong / R2 correct, n	R1 correct / R2 wrong, n	McNemar χ^2	P	BH-adjusted P
Mesencephalon	GPT-4o	N3	16	0	0		nan	nan
Mesencephalon	GPT-5	N3	16	0	0		nan	nan
Pons	GPT-4.0	GPT-o3 pro	77	0	52	50.02	<0.001	<0.001
Pons	GPT-4.0	N2	77	5	39	24.75	<0.001	<0.001
Pons	GPT-4.1	GPT-o3 pro	77	0	30	28.03	<0.001	<0.001
Pons	GPT-4o	GPT-o3 pro	77	0	48	46.02	<0.001	<0.001
Pons	GPT-5	GPT-o3 pro	77	2	39	31.61	<0.001	<0.001
Pons	GPT-o3 pro	N1	77	34	2	26.69	<0.001	<0.001
Pons	GPT-4o	N2	77	4	34	22.13	<0.001	<0.001
Pons	GPT-4.0	N3	77	5	34	20.10	<0.001	<0.001
Pons	GPT-4.0	GPT-o3	77	6	35	19.12	<0.001	<0.001
Pons	GPT-o3	GPT-o3 pro	77	2	25	17.93	<0.001	<0.001
Pons	GPT-o3 pro	N3	77	26	3	16.69	<0.001	<0.001
Pons	GPT-4o	N3	77	5	30	16.46	<0.001	<0.001
Pons	GPT-4o	GPT-o3	77	7	32	14.77	<0.001	<0.001
Pons	GPT-4.0	GPT-4.1	77	5	27	13.78	<0.001	<0.001
Pons	GPT-o3 pro	N2	77	22	4	11.12	<0.001	.002
Pons	GPT-4.0	N1	77	8	28	10.03	.002	.003
Pons	GPT-4.1	GPT-4o	77	25	7	9.03	.003	.006
Pons	GPT-5	GPT-o3	77	4	18	8.91	.003	.006
Pons	GPT-4o	N1	77	6	22	8.04	.005	.009
Pons	GPT-5	N2	77	11	30	7.90	.005	.009
Pons	GPT-4.0	GPT-5	77	6	21	7.26	.007	.012
Pons	GPT-5	N3	77	7	21	6.04	.014	.023

Region	Rater 1	Rater 2	N	R1 wrong / R2 correct, n	R1 correct / R2 wrong, n	McNemar χ^2	P	BH-adjusted P
Pons	N1	N2	77	12	26	4.45	.035	.055
Pons	GPT-4.1	N2	77	11	23	3.56	.059	.089
Pons	GPT-4o	GPT-5	77	10	21	3.23	.072	.104
Pons	GPT-4.1	GPT-5	77	14	7	2.33	.127	.175
Pons	GPT-4.1	GPT-o3	77	8	15	2.13	.144	.193
Pons	GPT-o3	N1	77	20	11	2.06	.151	.194
Pons	N1	N3	77	12	21	1.94	.164	.203
Pons	GPT-4.1	N3	77	11	18	1.24	.265	.318
Pons	GPT-5	N1	77	8	13	1.19	.275	.320
Pons	GPT-4.0	GPT-4o	77	7	11	0.89	.346	.389
Pons	GPT-o3	N2	77	12	17	0.55	.458	.499
Pons	N2	N3	77	18	13	0.52	.472	.500
Pons	GPT-4.1	N1	77	15	13	0.04	.850	.874
Pons	GPT-o3	N3	77	15	15	0.00	1.000	1.000
