# Supplementary material for: Symptom-Only Localization of Brainstem Ischemia Using Large Language Models Versus Neurologists in Diffusion-Weighted Imaging–Positive Cases: Retrospective Single-Center Study
Source: JMIR Form Res. 2026 Jul 8;10:e87163. doi: 10.2196/87163 (PMC13345501; doi:10.2196/87163)
Supplement: Multimedia Appendix 4 [file formative-v10-e87163-s004.pdf]

Supplementary Table 3. Distribution of Predicted Anatomical-Lateral Classes Across Raters

Values are shown as percentage (n) of all 109 predictions per rater. Model names use hyphenated notation.

Rater	Left midbrain	Right midbrain	Left pons	Right pons	Left medulla	Right medulla	Invalid/ missing
GPT-4.0	1.8% (2)	1.8% (2)	33.0% (36)	46.8% (51)	3.7% (4)	2.8% (3)	10.1% (11)
GPT-4.1	4.6% (5)	8.3% (9)	32.1% (35)	29.4% (32)	12.8% (14)	11.0% (12)	1.8% (2)
GPT-4o	2.8% (3)	8.3% (9)	35.8% (39)	45.0% (49)	5.5% (6)	2.8% (3)	0% (0)
GPT-5	7.3% (8)	11.9% (13)	30.3% (33)	34.9% (38)	5.5% (6)	9.2% (10)	0.9% (1)
GPT-o3	28.4% (31)	7.3% (8)	25.7% (28)	20.2% (22)	11.0% (12)	7.3% (8)	0% (0)
GPT-o3 pro	88.1% (96)	0.9% (1)	5.5% (6)	2.8% (3)	1.8% (2)	0.9% (1)	0% (0)
N1	9.2% (10)	9.2% (10)	40.4% (44)	20.2% (22)	4.6% (5)	16.5% (18)	0% (0)
N2	4.6% (5)	9.2% (10)	16.5% (18)	22.0% (24)	24.8% (27)	22.9% (25)	0% (0)
N3	10.1% (11)	10.1% (11)	21.1% (23)	24.8% (27)	19.3% (21)	14.7% (16)	0% (0)
nan	nan	nan	nan	nan	nan	nan	nan
